# *FDXR* variants cause adrenal insufficiency and atypical sexual development

**DOI:** 10.1172/jci.insight.179071

**Published:** 2024-06-17

**Authors:** Emanuele Pignatti, Jesse Slone, María Ángeles Gómez Cano, Teresa Margaret Campbell, Jimmy Vu, Kay-Sara Sauter, Amit V. Pandey, Francisco Martínez-Azorín, Marina Alonso-Riaño, Derek E. Neilson, Nicola Longo, Therina du Toit, Clarissa D. Voegel, Taosheng Huang, Christa E. Flück

**Affiliations:** 1 Division of Pediatric Endocrinology, Diabetology and Metabolism, Department of Pediatrics, Inselspital, Bern University Hospital, and; 2 Department of Biomedical Research, University of Bern, Bern, Switzerland.; 3 Department of Pediatrics, Jacobs School of Medicine and Biomedical Sciences, University at Buffalo, Buffalo, New York, USA.; 4 Department of Pediatrics, Endocrinology Unit, and; 5 Unidad de Dismorfología y Genética (UDISGEN), 12 de Octubre University Hospital, Madrid, Spain.; 6 Grupo de Enfermedades Raras, Mitocondriales y Neuromusculares (ERMN), Instituto de Investigación Hospital 12 de Octubre (imas12), E-28041 Madrid, Spain.; 7 Centro de Investigación Biomédica en Red de Enfermedades Raras (CIBERER), U723, E-28041 Madrid, Spain.; 8 Pathology Department, 12 de Octubre University Hospital, Madrid, Spain.; 9 Division of Genetics and Metabolism, Department of Child Health, The University of Arizona College of Medicine, Phoenix, Arizona, USA.; 10 Division of Medical Genetics, Department of Pediatrics, University of Utah, Salt Lake City, Utah, USA.; 11 Department of Nephrology and Hypertension, Inselspital, Bern University Hospital, University of Bern, Bern, Switzerland.

**Keywords:** Endocrinology, Genetics, Genetic diseases, Mitochondria, Molecular genetics

## Abstract

Genetic defects affecting steroid biosynthesis cause cortisol deficiency and differences of sex development; among these defects are recessive mutations in the steroidogenic enzymes CYP11A1 and CYP11B, whose function is supported by reducing equivalents donated by ferredoxin reductase (*FDXR*) and ferredoxin. So far, mutations in the mitochondrial flavoprotein FDXR have been associated with a progressive neuropathic mitochondriopathy named FDXR-related mitochondriopathy (FRM), but cortisol insufficiency has not been documented. However, patients with FRM often experience worsening or demise following stress associated with infections. We investigated 2 female patients with FRM carrying the potentially novel homozygous FDXR mutation p.G437R with ambiguous genitalia at birth and sudden death in the first year of life; they presented with cortisol deficiency and androgen excess compatible with 11-hydroxylase deficiency. In addition, steroidogenic *FDXR*-variant cell lines reprogrammed from 3 patients with FRM fibroblasts displayed deficient mineralocorticoid and glucocorticoid production. Finally, *Fdxr*-mutant mice allelic to the severe p.R386W human variant showed reduced progesterone and corticosterone production. Therefore, our comprehensive studies show that human *FDXR* variants may cause compensated but possibly life-threatening adrenocortical insufficiency in stress by affecting adrenal glucocorticoid and mineralocorticoid synthesis through direct enzyme inhibition, most likely in combination with disturbed mitochondrial redox balance.

## Abstract 2

Human ferredoxin reductase variants may cause a progressive neuropathic mitochondriopathy but also can cause adrenal insufficiency and genital ambiguity.

## Introduction

Cortisol is the most important hormone for surviving acute stress related to severe life-threatening events such as infections. So far, genetic variants have been described in humans for all enzymes involved in adrenal steroidogenesis leading to congenital adrenal hyperplasia (CAH) defined by cortisol deficiency and lack or excess of androgen production depending on the underlying specific defect and the chromosomal sex. Cortisol deficiency has also been associated with variants in the genes for steroidogenic acute regulatory (STAR) protein, which transports cholesterol into the mitochondria, and for the redox partner P450 oxidoreductase (*POR*) (1, 2).

**Figure 1:**
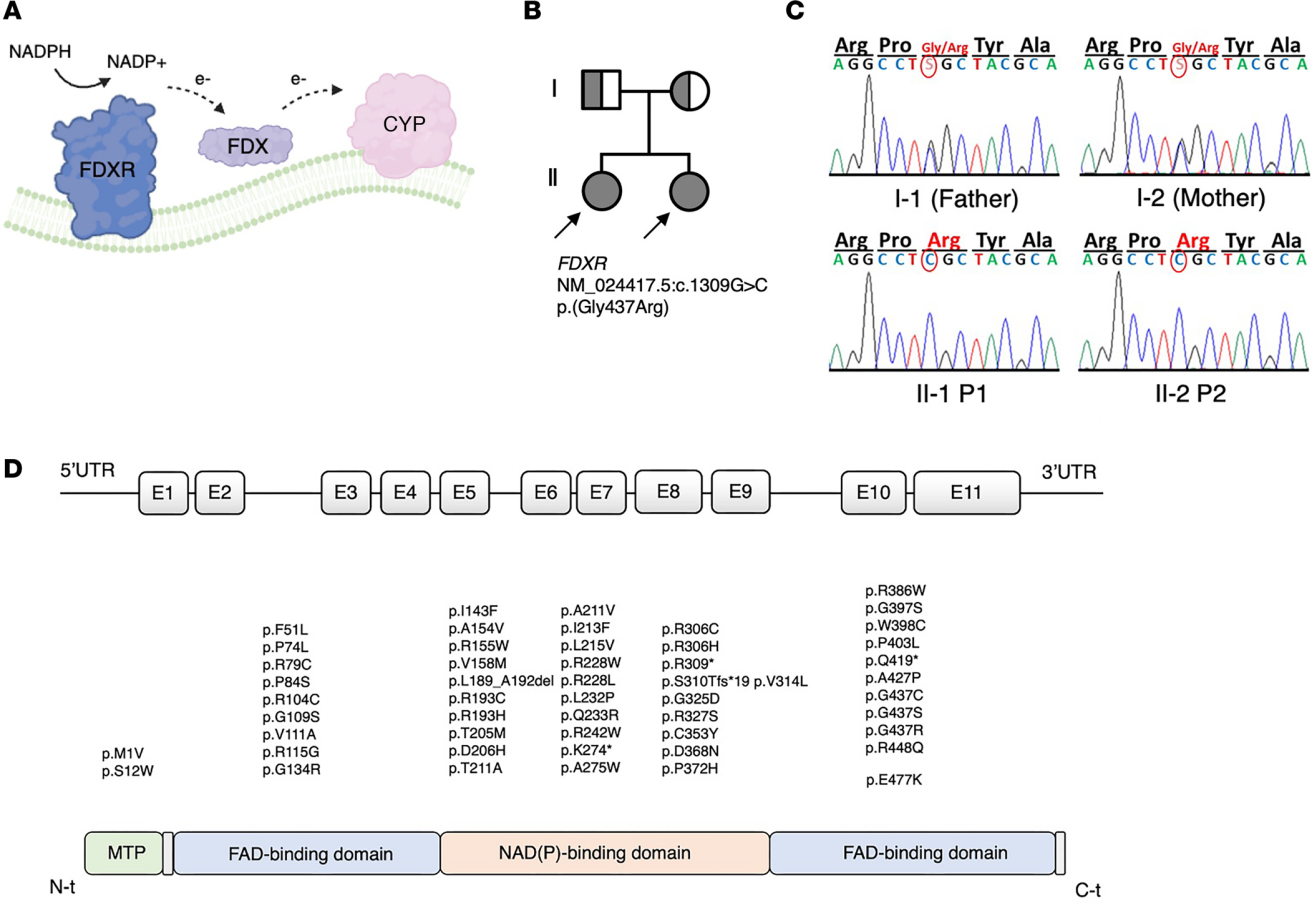
Role of FDXR and genetic characteristics of the *FDXR* variants identified in the index patients and of the reported patients manifesting with FDXR-related mitochondriopathy (FRM). (**A**) Schematic representation of the role of the flavoprotein ferredoxin–NADP(+) reductase (FDXR) as electron acceptor from nicotinamide adenine dinucleotide phosphate (NADPH), and electron donor for ferredoxin proteins (FDX), from where electrons are finally donated to effector Cytochrome P450 (CYP) enzymes associated to the inner mitochondrial membrane. (**B**) Pedigree of a family in which the 2 daughters are affected by neuropathy and adrenal insufficiency caused by the homozygous c.1309G > C (p.G437R) variant in *FDXR*. (**C**) displays the result of Sanger sequencing around the c.139 region for the members of the family in **B**. (**D**) reviews the *FDXR* variants that have been described in patients with FRM as of June 2023, including the novel p.G437R described in the index patients in this manuscript, aligned to the relevant protein domain. Domain annotation is based on a crystallography analysis of the *Bos taurus* FDXR ortholog (36).

All cytochrome P450 (CYP) steroid enzymes involved in cortisol production depend on redox partners for electron transfer (3). POR supports all type 2 microsomal CYP enzymes located in the endoplasmic reticulum, including CYP17A1, CYP21A2, and CYP19A1 for steroid biosynthesis. By contrast, type 1 CYP enzymes located in the mitochondria obtain reducing equivalents sequentially from the flavoprotein ferredoxin–NADP(+) reductase (FDXR) and the ferredoxins (FDX) (Figure 1A). Among the mitochondrial type 1 enzymes, CYP11A1 and CYP11B1 are essential for cortisol production, and CYP11B2 catalyzes aldosterone biosynthesis. Although loss of activity of these enzymes leads to cortisol and/or aldosterone deficiency, variants in human *FDXR* or *FDX* have not been associated with adrenal disease so far. However, inactivation of the related *Fdx1b* gene in zebrafish and the *dare* gene in *Drosophila* (an ortholog of human *FDXR*) results in defective steroid production, suggesting that FDXR/FDX deficiency may lead to impaired steroidogenesis in humans (4–6). FDXR is normally not rate limiting for the activities of mitochondrial CYP enzymes, but mutagenesis of FDXR has been shown to affect the steroidogenic CYP reactions (7) and also showed higher K_m_ and lower V_max_ values for the reduction of FDX.

In 2017, autosomal recessive *FDXR* mutations were first reported in patients suffering from a potentially novel mitochondriopathy manifesting with optic atrophy and neuropathy (8, 9). As of June 2023, 77 patients have been described with numerous *FDXR* variants spread throughout the gene, mostly missense mutations carried either in homozygosity or in compound heterozygosity (Supplemental Table 1; supplemental material available online with this article; https://doi.org/10.1172/jci.insight.179071DS1) (8–18). The disease spectrum comprises visual and hearing defects and a broad range of central and peripheral neuropathies. Affected individuals show variable degrees of disease severity, with one group manifesting early in life and often worsening over time, especially after intercurrent infections, and the other manifesting after the age of 2 years with milder disease course (19).

We now describe 2 siblings with severe biallelic *FDXR* mutations manifesting at birth with optic atrophy, neuropathic hearing loss, global encephalopathy, and a 46,XX androgen excess leading to variation of sex development combined with adrenal insufficiency. We confirm pathogenicity of human *FDXR* mutations on steroidogenesis by functional studies performed in patient-derived reprogrammed adrenal cell lines and a mouse model of the disease. Thus, our work adds *FDXR* variants to the list of genes that may cause adrenal insufficiency and a form of syndromic 46,XX CAH featuring androgen excess.

## Results

### Disrupted adrenal steroidogenesis in index patients with autosomal recessive FDXR variation.

Two Equatoguinean siblings presented at birth with ambiguous genitalia and were found to have a 46,XX androgen excess variation of sex development with normal uterus and ovaries. Soon after birth, they were also diagnosed with a severe sensorial neuropathy compatible with an optic atrophy–ataxia–peripheral neuropathy–global developmental delay syndrome. Adrenal insufficiency was suspected in follow-up visits (Table 1 and Supplementary Information). Both infants died in the first year of life due to infections and respiratory failure. Postmortem examination of the adrenals of index patient 2 revealed slightly heavier adrenal glands, with minimal cytoplasmic vacuolization indicating lipid overload (Supplemental Figure 3 and Supplementary Information).

Whole-exome sequencing (WES) analysis excluded variations in known CAH genes (such as *HSD3B2*, *CYP21A2*, *CYP11A1*, *CYP11B2*, *CYP11B1*, and *POR*) but found a potentially novel homozygous variant in the *FDXR* gene: c.1309G > C (p.G437R) (Figure 1, B and C). Both parents were healthy carriers.

### Characteristics of reported patients with biallelic FDXR variants pointing at impaired steroidogenesis.

Review of the literature revealed 77 patients with 59 biallelic *FDXR* mutations (Figure 1D) presenting with visual and hearing defects and a broad range of central and peripheral neuropathies (Supplementary Information) (10–18). Affected individuals show variable degrees of disease severity, with one group manifesting early in life and often worsening with intercurrent infections and the other manifesting after the age of 2 years with milder disease course (19). Many early-onset cases carried a specific variant (p.R386W) with high prevalence in the Mexican population (19). Investigating for signs and symptoms of potential undiagnosed adrenal insufficiency (Supplemental Table 1), we found a history of severe, often life-threatening events or deadly infections in 20 cases (26%). These events were mostly associated with a deterioration of preexisting signs of mitochondriopathy. Twelve patients died between 0.5 and 6 years, mostly following an infection. Interestingly, we also found 2 patients with a genital phenotype. A boy was reported to have cryptorchidism and a micropenis (16), while a girl was noted to have labial fusion and clitoromegaly (13).

### Reprogrammed patient-derived adrenal cell lines carrying FDXR variants show impaired steroidogenesis.

**Figure 2:**
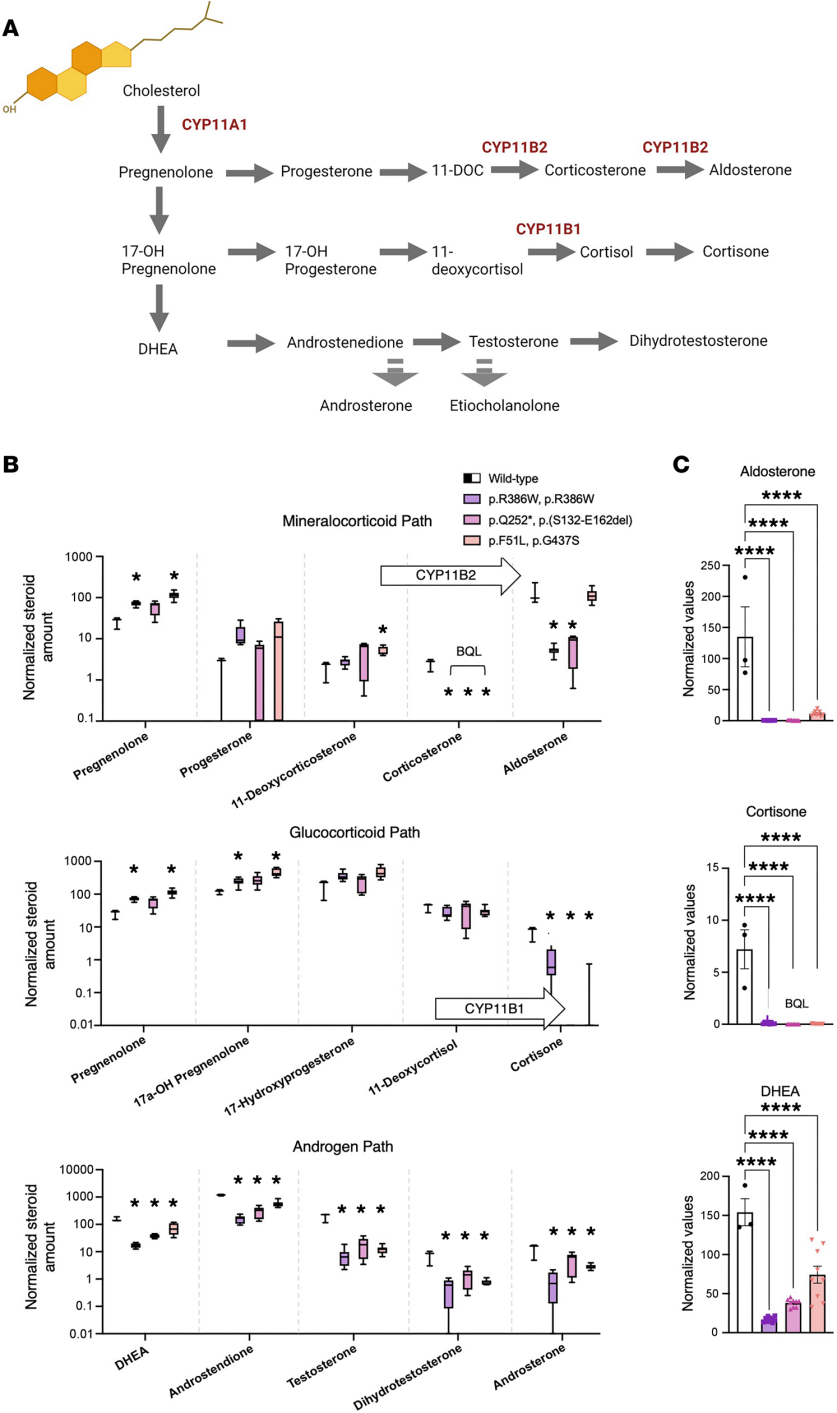
Cells from FDXR patient display low CYP11B1 and CYP11B2 enzymatic activity. (**A**) Classical steroids and steroidogenic pathways, all initiated from cholesterol (top left), occurring in the human adrenal cortex. In red, the official names of the 3 FDXR-dependent mitochondrial steroidogenic enzymes, namely CYP11A1 (also known as Cholesterol side-chain cleavage enzyme), CYP11B2 (Aldosterone Synthase), and CYP11B1 (Steroid 11β-hydroxylase). (**B**) Steroid amounts in culture media conditioned by reprogrammed fibroblasts from FDXR patients compared with control values (representing steroid amounts in culture media conditioned by reprogrammed fibroblasts from a single nonaffected individual; i.e., Control). Steroids are split among 3 graphs according to their belonging to a specific steroid class. Arrows containing the names of enzymes indicate the enzymatic reaction carried out by the enzyme. Asterisks reflect discoveries found using a multiple unpaired *t* test assuming individual variance for each steroid. *P < 0.05. (**C**) The endpoint or most representative steroids for each pathway on the left, on a linear scale. Statistical analysis was conducted using a 1-way ANOVA followed by Dunnett’s multiple comparisons test. All values in **B** and **C** are normalized by *GAPDH* transcripts contained within the cell monolayer, used as a proxy for cell number, as reported in Supplemental Figure 1D. Below quantification level (BQL) indicates the samples in which steroid levels were not measurable above the lowest quantification limit using LC-MS. DHEA, Dehydroepiandrosterone; 11-DOC, 11-deoxycorticosterone. ****P_adj_ < 0.0001.

To gain further insight into the effect of FDXR variants on steroidogenesis, we reprogrammed 3 available dermal fibroblast lines originating from male patients into induced adrenal-like cell (iALC) lines and assessed their steroid production along the 3 classical pathways of mineralocorticoid, glucocorticoid, and androgen biosynthesis (Figure 2A). The patient with the most severe disease phenotype (male patient II-2 in ref. 9, diagnosed with delays in motor development and poor visual tracking at 3 months of age and died at 17 months) carried a homozygous mutation in the hotspot p.R386W. The other 2 patients, Case 2 in ref. 9 (p.F51L/p.G437S), and Case 14 in ref. 19 (p.Q252*, p.S132-E162del), both males, had a later onset of neurological disease at age 2 and 4 years, respectively (Supplemental Table 1). While previous studies show reduced FDXR expression in these patients’ fibroblasts (9, 19), FDXR, FDX1, and FDX2 RNA expression in the corresponding iALC was not significantly different compared with WT iALC (Supplemental Figure 2). Steroid profiles of all 3 cell lines show reduced corticosterone, cortisone, and androgen production in comparison with a sex-matched control line carrying a fully functional FDXR (Figure 2, B and C). Cortisone was measured as a proxy for cortisol, which was below the lowest accurate quantification threshold. Aldosterone was reduced in 2 mutant lines, 1 corresponding to the most severe patient. Altogether, these data show that *FDXR* variants inhibit CYP11B2 and CYP11B1 enzymatic activities. In addition, the suppression of the androgen pathway (Figure 2B) and the lower amounts of total steroid output (Supplemental Figure 1E) point at a lower conversion of cholesterol into pregnenolone precursor by CYP11A1.

### In silico analysis of FDXR variants.

**Figure 3:**
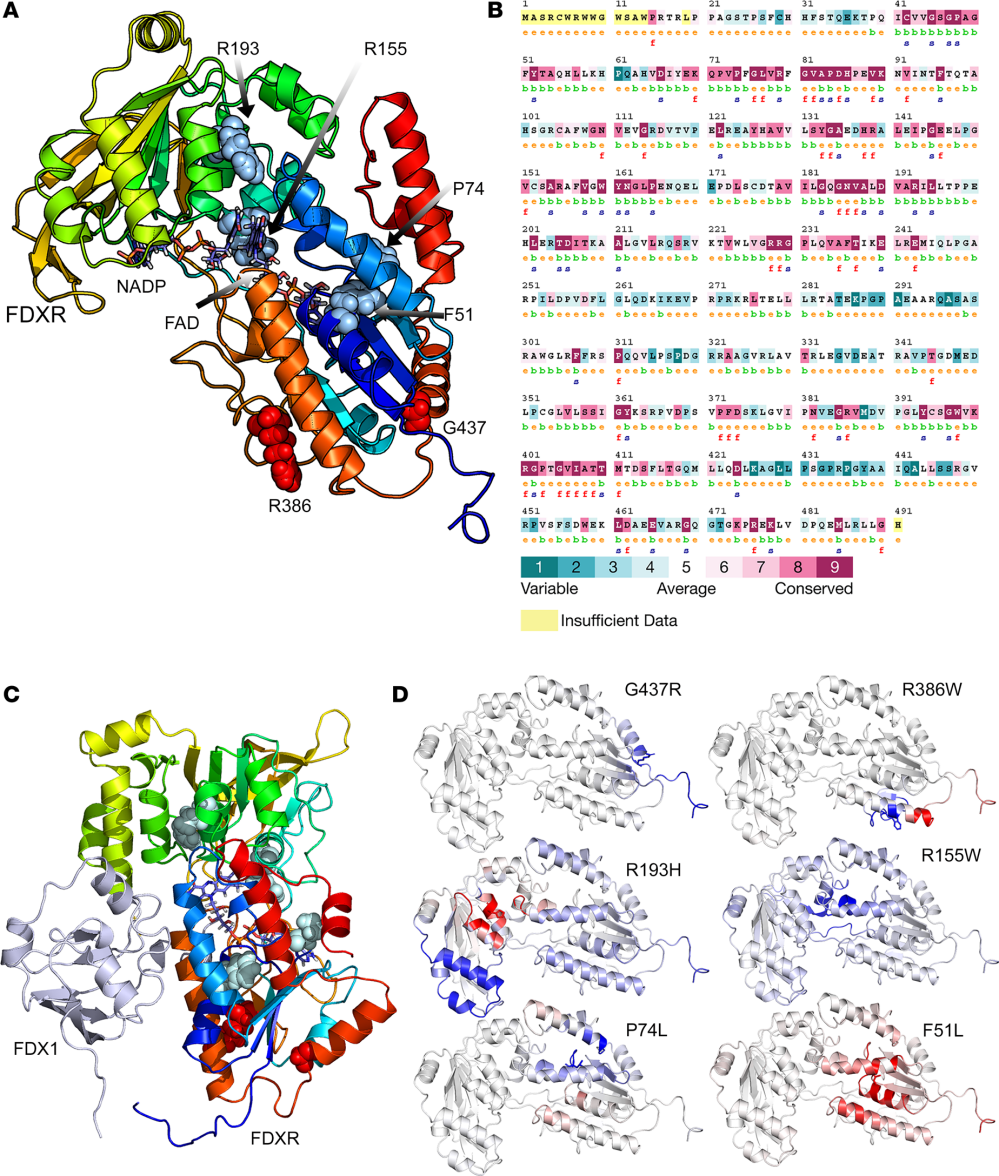
Sequence and structure analysis of mutations in FDXR. (**A**) A 3D model of human FDXR displayed as a ribbon diagram. The positions of the phenylalanine 51, proline 74, arginine 155, arginine 193, arginine 386, and glycine 437 residues are indicated. The structural model of human FDXR is based on a known 3D structure of the bovine protein as described in the Methods section. The diagram is colored using a rainbow palette with blue at N-terminus and red at C-terminus. Cofactors (NADP, FAD) are shown as stick models, while amino acids phenylalanine 51, proline 74, arginine 155, arginine 193, arginine 386, and glycine 437 are shown as spheres. (**B**) The evolutionary sequence conservation of FDXR. Most of the mutations reported in this study are highly conserved across species and are predicted to have structural roles. Sequences are colored based on amino acid conservation, with dark blue being the least conserved and dark red being the most conserved, while yellow indicates that no prediction could be made. (**C**) A complex of FDXR and FDX1 proteins showing the locations of mutated residues, which are not at the FDXR-FDX interface and are predicted not to have a direct effect on FDX-FDXR interaction. (**D**) Stability and flexibility analysis of mutated FDXR structures compared with WT FDXR. An increased flexibility was observed for amino acid changes F51L and R193H (shown in red), indicating decreased stability that was supported by differential free energy calculations. Decreased flexibility due to P74L, R155W, R386W, and G537R mutations is shown in blue.

To understand how the variants in our index patients and in cells disrupt protein function, we performed computational modeling of the human FDXR structure (Figure 3A) and performed evolutionary analysis (Figure 3B) as well as interaction with FDX (Figure 3C) and structural flexibility studies (Figure 3D). All mutants studied in this report (in index patients and patient-derived cell lines) were predicted to have a negative effect — either by increased rigidity that affects electron transfer within the FDXR from reduced nicotinamide adenine dinucleotide phosphate (NADPH) to flavin adenine dinucleotide (FAD) or via increased flexibility that affects the interaction with FDX as well as the fine balance of intramolecular movements that allow electron transport from NADPH to FAD and binding and release of NADPH/NADP (Figure 3D, Supplemental Table 2, and Supplementary Information).

The presence of any of the described mutations in compound heterozygous form with other structural mutants or mutations resulting in a truncated protein or nonsense mediated RNA decay are predicted to have severe disease–causing effects. We also built models of human FDXR in complex with human FDX1 (Figure 3D) and found that none of the mutants described in the current study were located at or near the FDXR-FDX interface. These studies are in agreement with published crystal structures of the FDXR-FDX complex (20, 21) as well as a solution model based on paramagnetic NMR spectroscopy (7). Keizers et al (7) created mutants of FDXR and found reduced affinity of FDX as well as lower activities of steroidogenic P450 CYP11A1. Crystal structure of the FDXR-FDX complexes as well as a paramagnetic NMR model of the complex agree with our docked structures, which indicate a role of electrostatic interactions in the electron transfer between FDXR and FDX. A direct effect on any cytochrome P450 could not be predicted due to an indirect effect of FDXR interactions with the steroidogenic CYP activities. Nonetheless, due to the predicted variable nature of protein-protein interactions resulting from different conformational changes, a variable effect on the ability of FDX proteins to be reduced by mutant FDXR proteins is predicted.

### A mouse model of the R386W hotspot mutation shows no alteration of adrenal development and zonal organization, but diminished corticosterone synthesis.

**Figure 4:**
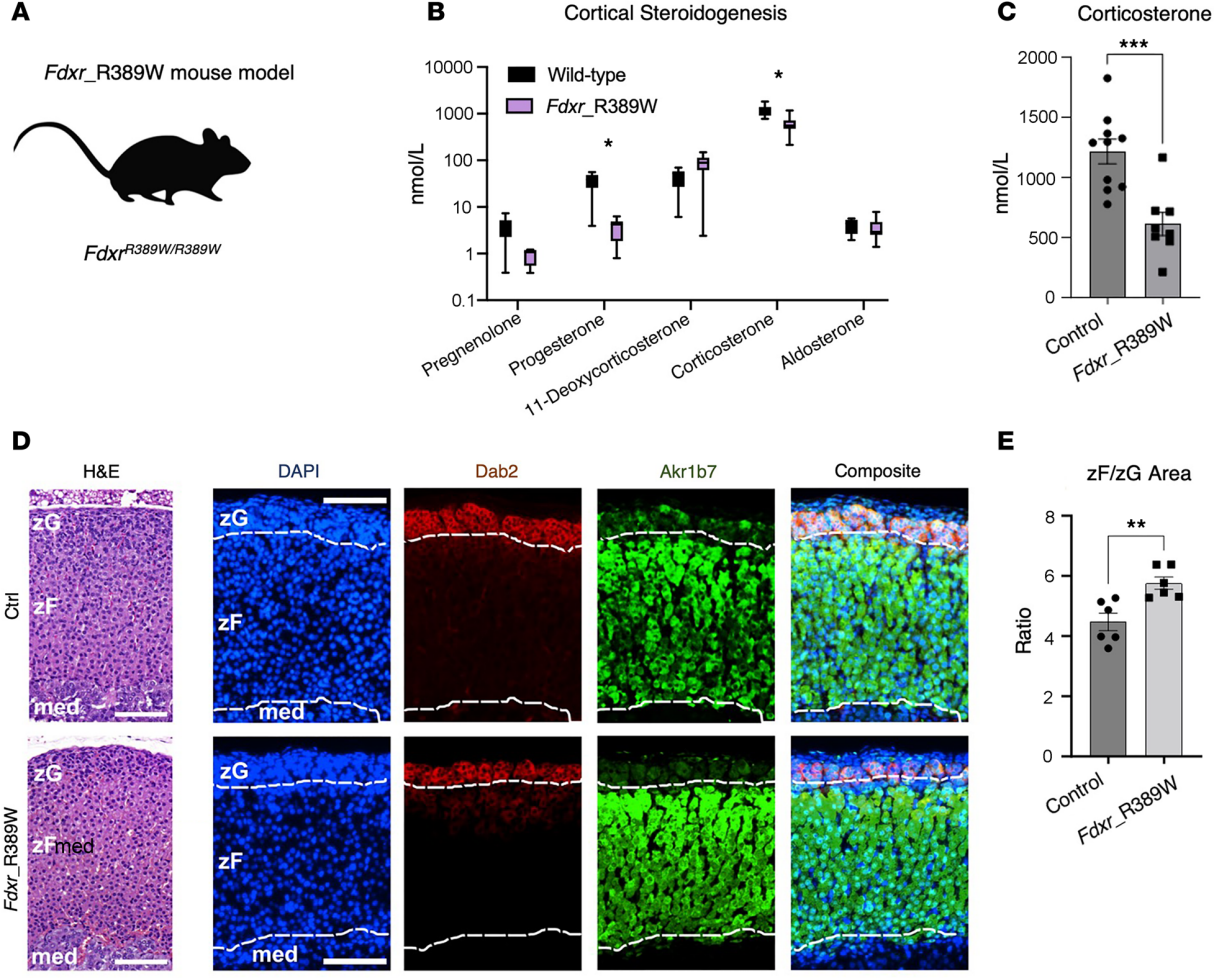
The *Fdxr*^R389W^ mouse model shows no impairment of adrenal structure and zonation. (**A**) Schematic of the novel mouse model (*Fdxr*^R389W^) carrying homozygous R389W mutations, allelic to the hotspot R386W variant in FDXR patients. (**B**) Serum steroid profile of the *Fdxr*^R389W^ mice compared with control animals. Asterisks reflect discoveries found using a multiple unpaired *t* test assuming individual variance for each steroid, with FDR, and a 2-stage step-up method (Benjamini, Krieger, and Yekutieli). *P < 0.01. (**C**) Serum levels of corticosterone, the main glucocorticoids in mice, in control and *Fdxr*^R389W^ mice. Significance was tested using an unpaired *t* test. (**D**) Micrographs of representative adrenal sections, either stained with H&E (left) or immunoassayed with Dab2 (zona glomerulosa, zG), Akr1b7 (zona fasciculata, zF), and DAPI (for nuclei; right panels). Scale bar: 200 μm. Dotted white lines outline the zG region as identified using Dab2 staining, and the corticomedullary (med) region (below) as marked by the lower boundary of the Akr1b7 staining. (**E**) Ratio values calculated as zF area normalized by zG area, measured on 6 independent entire adrenal coronal sections for either controls or *Fdxr*^R389W^ samples. An unpaired *t* test was used to calculate significance. **P < 0.01; ***P < 0.001.

To assess whether *FDXR* variants in affected patients affect adrenocortical development, zonation, and steroidogenic function, we used a mouse model of the disease carrying the p.R389W mutation (*Fdxr*^R389W^), allelic to the human p.R386W hotspot mutation characterized in patients (Figure 4A). This mouse model has been generated in Jesse Slone’s and Taosheng Huang’s lab at University at Buffalo. Steroid profiling of mouse serum showed decreased corticosterone levels, suggesting that FDXR supports CYP11B1 activity in mice, too (Figure 4, B and C). Histological analysis aided by zone-specific staining (Dab2 to identify the zona glomerulosa [zG], and Akr1b7 to identify the zona fasciculata [zF]) did not reveal any major defect of tissue organization or zonal arrangement (Figure 4D). Only the ratio between the areas occupied by the outer cortical zones (zG, producing aldosterone; zF, producing corticosterone-) indicated an expansion of the zF at the expense of the zG (Figure 4E), which is compatible with chronically elevated ACTH due to primary adrenal insufficiency (22).

## Discussion

Here, we show that biallelic *FDXR* variants may cause lethal adrenocortical insufficiency and a 46,XX variation of sex development. This has not been recognized to date, despite FDXR being an essential reducing partner to 3 steroid producing CYP enzymes (CYP11A1, CYP11B1, and CYP11B2).

In the human adrenal cortex, cholesterol is converted to glucocorticoids and mineralocorticoids through a series of enzymatic reactions (Figure 2A), whose disruption results in a range of cortisol insufficiencies cataloged as CAH. Additionally, precursor accumulation and diversion to the adrenal androgen pathway in 46,XX CAH may lead to androgen excess and consequent prenatal virilization of females with autosomal recessive *HSD3B2*, *CYP21A2*, *CYP11B1*, and *POR* mutations. Typical of CYP11B1 deficiency is an increase in blood pressure with aging caused by the accumulation of precursors with mineralocorticoid activity (23). The FDXR index patients reported here displayed both ambiguous genitalia and elevated blood pressure, which recapitulate CAH due to deficiency of CYP11B1. Decreased glucocorticoids in the adrenal-like *FDXR* variant cell lines and the *Fdxr* mouse were consistent with index patients’ laboratory findings (Table 1), with reduced mineralocorticoid production additionally evident in the in vitro models.

Cortisol deficiency with POR deficiency, the obligate redox partner of all type 2 endoplasmic P450s enzymes (24), is only clinically relevant upon severe stress, while its effect on sex steroids is often most prominent, manifesting with a wide spectrum (25). Similarly, our index patients with *FDXR* variants had adrenocortical insufficiency, which might be subclinical and may become life threatening with infections triggering an adrenal crisis. However, whether the large spectrum of stress responses in FDXR patients can be explained either by variant genotype or other mechanisms such as cortisol deficiency needs to be established. It is important to note that mitochondrial diseases are generally associated with increased susceptibility to infections, in some cases associated with reduced production and function of immune cells (26–28).

The steroid profile of our index 46,XX patients, which includes androgen excess, suggests that CYP11A1 enzyme activity is less affected by FDXR insufficiency than CYP11B1. This might be explained by the fact that the electron transport chain for CYP11A1 is relatively conservative, losing only 15% of electrons, in contrast to a 40% loss associated with the CYP11B1 reaction, indicating that CYP11B1 may be more affected by an inefficient FDXR/FDX system (29). Additionally, an analysis of CYP11/FDX complexes by surface plasmon resonance (SPR) indicated that the association constant for the CYP11A1/FDX complex was higher compared with the CYP11B1/FDX and CYP11B2/FDX complexes (30). This suggests a competition for available reduced FDXR between CYP11A1/FDX and CYP11B1/FDX complexes which favors the CYP11A1-mediated reaction. Furthermore, the SPR data indicate that the CYP11A1/FDX/FDXR complex is enthalpy driven while CYP11B/FDX/FDXR complexes are entropy driven, also indicating that the intrinsic disorder in FDXR interactions may favor the CYP11A1 activity over CYP11B1. However, additional studies using multiple combinations of WT and mutant FDXR proteins with FDX and different steroidogenic CYP proteins are needed to further characterize the effect of mutations in FDXR on individual CYP reactions and overall steroidogenesis.

CAH can also be associated with mutations in the STAR protein, responsible for importing cholesterol into mitochondria. Interestingly, STAR activity is inversely regulated by changes of the mitochondrial redox homeostasis, which is strongly affected in patients with FRM (13), suggesting that STAR activity may also be compromised (14, 31). STAR deficiency results in lipoid CAH, which presents as a 2-hit model disease: first, cholesterol transport into mitochondria is reduced; second, the progressive accumulation of cholesterol depots results in cell death (32). We suspect that FDXR-associated adrenocortical insufficiency could also be a progressive 2-hit adrenal disorder, where the first genetic hit causes reduced electron transfer to dependent steroidogenic enzymes, while the second hit consists of accumulation of reactive oxygen species and disruption of oxidative phosphorylation associated with FDXR’s role in Fe-S cluster assembly, inevitably causing cell death (9, 14). Despite that, our *Fdxr* mouse model showed an almost normal cortex with only a slight expansion of the zF at the expense of the zG. This discrepancy may be due to the young age at which the animal tissues were collected, and this prevented us from exploring long-term consequences of FDXR inactivation.

A recent systematic review concluded that adrenal insufficiency is seldom seen with mitochondrial disorders, although mitochondria play an important role for cortisol synthesis not only by harboring part of the steroid biosynthesis machinery but also through ATP generation and ROS detoxification (31, 33). However, subclinical cortisol deficiency was more often observed in patients with mitochondrial diseases (e.g., Pearson syndrome and Kearns-Sayre syndrome) when assessed by ACTH stimulation tests or with longitudinal evaluation (34). By contrast, primary adrenal insufficiency is a major phenotype of the peroxisomal disorder adrenoleukodystrophy (ALD), in which very long–chain fatty acids accumulate and lead to cytotoxic destruction of the adrenal cortex, while a disturbed redox homeostasis is also involved (35, 36). Like ALD, FDXR-related mitochondriopathy might affect steroidogenesis more often and more severely than other mitochondrial disorders, presumably because of combined disruption of steroid enzyme activity and redox homeostasis (2-hit model). In fact, previous mechanistic studies addressing the effect of human FDXR variants on redox potential in patients’ fibroblasts have shown significantly increased ROS production and decreased mitochondrial membrane potential relative to normal fibroblasts, likely due to the excessive buildup of mitochondrial iron in FDXR patient cells (9). Moreover, genetic variants of *NNT* and *TXNRD2*, which are involved in maintaining the mitochondrial ROS balance, have been shown to be disease causing in several patients with familial glucocorticoid deficiency informing the importance of the mitochondrial ROS system for steroidogenesis (37, 38).

In conclusion, adrenal insufficiency might be a fatal, hidden threat to patients with FRM for which they should be screened and treated as indicated. *FDXR* variants seem to inhibit 11-hydroxylase activity predominantly. Therefore, adrenal disease of FRM can also manifest with inadequate cortisol production, increased blood pressure, and in 46,XX patients with ambiguous genitalia at birth or hirsutism/androgen excess later in life.

## Methods

### Sex as a biological variable.

The effect of FDXR variants in humans has been explored in 46,XX individuals (index patients) and in reprogrammed dermal fibroblasts obtained from 46,XY patients. While no investigation of sex as a biological variable can be conducted because of the different methodological approaches (clinical observation vs. steroidogenic profiling in cell) that prevent direct statistical analysis, we confirmed the effect of FDXR mutations on both sexes. Mouse experiments were conducted exclusively in males to reduce the variability linked to the estrous cycle and cannot predict whether females will recapitulate the phenotype.

### Genetic workup of the index patients.

For WES, the amplified DNA fragments were hybridized to the Agilent SureSelect Human All Exon V4 (51 Mb), the captured library was sequenced on a HiSeq 2000 platform, and the reads were aligned against the human reference genome (GRCh37 at UCSC) to obtain candidate variants. Nuclear variants and indels were prioritized according to the following criteria: (a) variants that were rare in healthy individuals (allele frequency below 0.01 for recessive or below 10−5 for dominant model of pathogenesis) or new (not described within public databases); (b) variants predicted to modify protein function (nonsense, splice site, coding indel, or missense variants); (c) variants consistent with a recessive model of pathogenesis: homozygous variants or 2 heterozygous variants present in the same gene; or (d) variants consistent with a dominant model of pathogenesis: heterozygous variants. Additional indications were obtained by using predictive software. Sanger sequencing of the candidate gene variant was performed for the patients and the parents. All sequences in this manuscript are annotated using the NM_024417.5 transcript variant.

### In silico analysis.

Initial analysis using Consurf included multiple sequence alignment of human FDXR with homologs from other species to determine sequence homology and conservation at amino acid substitution sites (39).Polymorphism Phenotyping v2 (PolyPhen-2) was then used to qualitatively predict the potential effect of the amino acid substitutions on the structure and function of the FDXR protein (40). The 3D models of human WT (Uniprot: P22570, NCBI: NP_077728) and variant FDXRs were constructed using previously published model-building protocols based on the crystal structure of bovine FDXR to study the potential effect of mutations on structure (36). Five different x-ray crystal structures of FDXR (PDB: 1E1N, 1CJC, 1E1K, 1E1M and 1E6E) were used to generate models of human FDXR, and they were then combined to generate a hybrid model retaining the best parts of individual structures. The modeled structure was then used to understand the structural basis of changes caused by the specific F51L, P74L, R155W, R193H, R386W, and G437R mutations.

The selection of templates was based on BLAST alignment scores, the WHAT_CHECK quality score (41) in the PDBFinder2 database (42), and the target coverage. For alignment correction and loop modeling, a secondary structure prediction for the target sequence was obtained by running PSI-BLAST to create a target sequence profile and feeding it to the PSIPRED secondary structure prediction algorithm (43). The stability of mutant proteins was analyzed using DUET (44), mCSM (45), and DynaMut2 (46) and by structural analysis of WT and mutant proteins running as a Python script under Yasara (47).

### Studies in reprogrammed cells.

Human fibroblasts were available for additional studies from previous work and originate from patients II-2 and 2 described in ref. 9, and patient 14 from ref. 19. They carry, respectively, the homozygous *FDXR* variant c.1156C > T, the c.151T > C and c.1309G > A variants in heterozygosity, and the c.736C > T and c.339G > A variants in heterozygosity. Fibroblasts were cultured in DMEM supplemented with 10% FCS (Invitrogen). Deprogramming to induced pluripotent stem cells (iPSC) followed a transgene-free modified Yamanaka protocol (48). Healthy sex-matched control iPSC were available from previous projects (49). Reprogramming to adrenal cortex-like cells was performed for both the healthy control line and the 3 iPSC lines carrying *FDXR* variant according to the Papadopoulos protocol (50). Briefly, a lentivirus was used to induce the expression of NR5A1, parallel to the exposure to dibutyryl cyclic adenosine monophosphate (dbcAMP), desert hedgehog (DHH), and human chorionic gonadotropin (hCG). Two weeks after NR5A1 transduction, steroid metabolites in the cell supernatant were measured by liquid chromatography coupled with mass spectrometry (LC-MS). Six technical replicates were carried over for each mutant cell line, while 2 replicates were used for the control line. In addition, 3 biological replicates were performed in all cases. The reprogrammed lines carrying a hypofunctional FDXR displayed a slower growth curve compared with the control line (Supplemental Figure 1A); therefore, raw data expressed in nmol/L (Supplemental Figure 1, B and C) were normalized by the cell content of *GAPDH* transcripts (Supplemental Figure 1D), used as a proxy for cell number, resulting in the normalized values in Figure 2B that are discussed here.

### Studies in Fdxr^R389W^ mice.

C57BL/6N mice carrying a single-point mutation in the *Fdxr* gene (c.1165 C > T), referred to within this work as *Fdxr*^R389W^ mice, were generated using CRISPR/Cas9 gene editing. The genotypes of the knock-in mice were confirmed using PCR and Sanger sequencing, and homozygous *Fdxr*^R389W/R389W^ mutant and *Fdxr*^+/+^ control mice were generated by crossing heterozygous *Fdxr*^R389W/+^ breeders. For ACTH stimulation testing, age-matched male *Fdxr*^R389W/R389W^ mutant and *Fdxr*^+/+^ control mice between 4 and 6 weeks of age were selected. ACTH stimulation was achieved using i.p. injection of 200 μg human ACTH (MilliporeSigma, A0298) into each mouse on the day of blood and tissue collection. Injections were always performed at the same time in the afternoon (around 3 p.m. local time) to avoid variation due to circadian rhythms. Mice were sacrificed at 1 hour after ACTH injection via CO_2_-mediated euthanasia, and blood was immediately collected via heart puncture. Adrenal gland tissues were also collected from each mouse for subsequent histological analysis. To obtain serum samples, the blood samples collected from each mouse were allowed to incubate for 30 minutes at room temperature and were then spun for 10 minutes at 2,000*g*. The serum fraction was then collected from the supernatant and frozen on dry ice before being placed at –80°C for long-term storage.

### Immunofluorescence and microscopy.

Adrenals were dissected from *Fdxr*^R389W^ male mice and strain-, age-, and sex-matched controls; cleared of the surrounding fat tissue; and fixed overnight in 4% PFA. Paraffin sections (5 μm) were processed for protein immunodetection as previously described (51). Briefly, antigen retrieval was carried out in 10 mM sodium citrate (pH 6.0), followed by overnight incubation with a mouse monoclonal anti–Disabled-2/p96 (Dab2; BD Transduction Laboratories, 610464) and a rabbit polyclonal anti-Akr1b7 (donated by Pierre Val and Antoine Martinez) (52). Indirect staining was performed using the goat anti–rabbit IgG (H+L) highly cross-adsorbed secondary antibody conjugated with Alexa Fluor 488, and a goat anti–mouse IgG (H+L) cross-adsorbed secondary antibody conjugated with Alexa Fluor 647 (both from Thermo Fisher Scientific; A11008 and A21235, respectively). DAPI was used for nuclear counterstaining. Images were captured using a Nikon Eclipse Ti-E upright microscope. H&E staining was carried out on neighboring sections with respect to the adrenal-matched immunofluorescence experiment. All H&E staining experiments in this manuscript were conducted according to standard protocols.

### Steroid profiling.

Steroid metabolites in the serum of *Fdxr*^R389W^ and control mice, as well as in media from reprogrammed patient cells, were measured by an established in house LC-MS method (53). Briefly, samples were collected and stored at –20°C until LC-MS analysis. The samples were purified using a solid-phase extraction on an OasisPrime HLB 96-well plate using a positive-pressure 96-well processor (both from Waters). For LC-MS analysis, a Vanquish UHPLC (equipped with an ACQUITY UPLC HSS T3 Column, 100 Å, 1.8 μm, 1 mm × 100 mm column; Waters) was coupled to a Q Exactive Plus Orbitrap (both from Thermo Fisher Scientific). Separation was achieved using gradient elution over 11 minutes using water and methanol, both supplemented with 0.1 % formic acid (all from Sigma-Aldrich) as mobile phases. Data analysis was performed using TraceFinder 4.1 (Thermo Fisher Scientific). The method was validated according to international standards, as previously described (51). Steroid hormone concentrations were calculated in nmol/L. As for data from cell media, values below quantification or detection thresholds were not used for statistics, unless all technical/biological replicates for a single condition had values below quantification threshold, in which case this was indicated in the plot as “below quantification level” by using the acronym BQL.

### Gene expression analysis.

RNA was purified from adrenal-reprogrammed cell monolayers using TRI Reagent (MilliporeSigma, T9424) and Direct-zol RNA kits (Zymo Research, R2051), following the manufacturer’s instructions. A complete protocol is provided in the Supplementary Information. RNA was reverse transcribed into cDNA using the High-Capacity cDNA Reverse Transcription Kit (Thermo Fisher Scientific, 4368814). Gene expression analysis was carried out by quantitative PCR (qPCR) using the QuantStudio 1 thermocycler (Invitrogen) and the PowerUp SYBR Green Master Mix (Thermo Fisher Scientific, A25780), according to manufacturer instructions. Technical duplicates were used to control for technical variability. The primers used for qPCR were: *GAPDH*: Fw, 5′-GCTCTCTGCTCCTCCTGTTC-3′; Rv, 5′-CGACCAAATCCGTTGACTCC-3′.

### Statistics.

Two-tailed Student’s *t* tests were used for comparisons between any 2 groups, while unpaired multiple 2-tailed *t* test was carried out when correcting for multiplicity of hypothesis testing unless otherwise specified in the figure legends. Statistical analysis was conducted using the Prism 10 software (GraphPad). The statistical details of the experiments can be found in the figure legends, whereby *n* values correspond to the number of independent samples. Data are presented as mean ± SEM; for box and whiskers plots, boxes extend from the 25th to 75th percentiles, the lines in the middle of the box are plotted at the median, and whiskers extend to the minimum and maximum values.

### Study approval.

Written informed consent was obtained from all patients and/or their parents participating in the study. Ethical approval for the studies of the index patients was from the Instituto de Investigación Hospital 12 de Octubre (i+12) in Madrid, Spain. Ethical approval for the studies with patients’ fibroblasts was consented by the IRBs of Cincinnati Children’s Hospital Medical Center (CCHMC) and State University at Buffalo. Ethical approval for mice studies came from the CCHMC and University at Buffalo IACUC.

### Data availability.

Data are available upon reasonable request from the corresponding authors subject to institutional review and approval. Values for all data points in graphs are reported in the Supporting Data Values file.

## Author contributions

EP performed experiments on mice biomaterials, analyzed data, created figures, and contributed to manuscript writing. JS generated and maintained the Fdxr mouse model, performed ACTH stimulation experiments on mice, and harvested mouse tissue and serum samples; he also contributed to manuscript writing and reviewing. EP and JS are co–first authors on this work for equal contributions, with EP put first for helping the last and corresponding authors in overall coordination of the study. MAGC identified and provided index patient information, performed clinical workup, and contributed to manuscript writing. TMC was involved in patient recruitment and in analysis and interpretation of genetic and metabolic data; TMC also contributed to manuscript writing and reviewing. JV performed ACTH stimulation experiments on mice and harvested mouse tissue and serum samples as well as contributed to manuscript reviewing. KSS performed experiments on human biomaterials and cell reprogramming, analyzed data, and contributed to manuscript writing. AVP performed bioinformatic structure and docking analyses and predictions as well as created related figures and text and contributed to manuscript writing and reviewing. FMA conducted the genetic analysis of index patients. MAR provided the histological analysis of index patients. DEN provided patient biomaterial and clinical and laboratory patient data. NL provided patient biomaterial and lab data. TDT and CV performed the steroid analysis of human and mouse biomaterials as well as participated in scientific discussion and manuscript writing and reviewing. TH contributed to the study idea and overall design and was PI of the mouse part of the project; he also provided patients’ fibroblast samples for experimental analysis and participated in manuscript writing and reviewing. CEF was the overall study PI and had the initial idea for the study; she was responsible for the overall study design, organization, data analysis and interpretation, preparation of figures and tables, manuscript writing, reviewing, and correspondence.

## Supplementary Material

Supplemental data

Supporting data values

## Figures and Tables

**Table 1:**
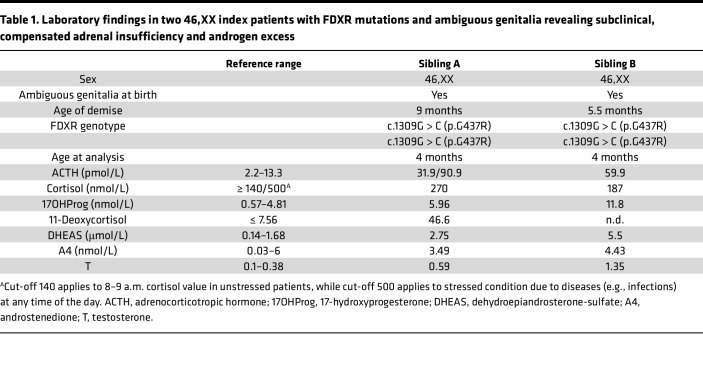
Laboratory findings in two 46,XX index patients with FDXR mutations and ambiguous genitalia revealing subclinical, compensated adrenal insufficiency and androgen excess

## Version 1 06/17/2024

In-Press Preview

## Version 2 07/22/2024

Electronic publication
