# Supplementary material for: FDXR variants cause adrenal insufficiency and atypical sexual development
Source: JCI Insight. 2024 Jun 17;9(14):e179071. doi: 10.1172/jci.insight.179071 (PMC11383170; doi:10.1172/jci.insight.179071)
Supplement: Supplemental data [file jciinsight-9-179071-s025.pdf]

Supplementary Information

Supplementary Appendix to Results and Methods Section

Detailed clinical data of index patients

Sibling A. The first daughter of this family was born prematurely at 36-3/7 weeks by induced vaginal delivery because of maternal preeclampsia. Her parents were healthy and non-consanguineous. Pregnancy was partially controlled in Equatorial Guinea but unremarkable. Birth weight and length were unknown. At birth, she presented with bilateral congenital cataracts and ambiguous genitalia. No history of prior spontaneous miscarriage, exposure to androgens or androgenic drugs during early development. Mother was on carbamazepine treatment because of seizures since adolescence. She was referred to the endocrinology clinic when she arrived in Spain at 4 months old. Her weight was 6.4 Kg (-0.2 SD), length 61 cm (-0.6 SD) and head circumference 39.5 cm (-1.8 SD). Physical examination revealed ambiguous genitalia with fusion of the labia majora, clitoromegaly and a common urogenital sinus (stage IV of Prader scale). She presented poor visual tracking, nystagmus and generalized hypotonia. There were no dysmorphic features or cutaneous abnormalities. Blood pressure was in the normal to high range for age (SBP 100 mmHg, 92nd percentile and DBP 50 mmHg, 90th percentile). Karyotype, microarray and *SRY* FISH were normal (46, XX, *SRY* negative). Abdominal and pelvic ultrasound scan (US) revealed hydrocolpos with lower vaginal atresia and normal-appearing adrenal glands, uterus, and ovaries. A retrograde genitography showed a 6 centimeter urethral structure and the bladder with absence of any fistula. Ophthalmology evaluation confirmed bilateral cataracts and small optic nerves. Brainstem evoked response audiometry demonstrated severe to profound bilateral sensorineural hearing loss. Full blood count, including lymphocyte subpopulations and immunoglobulin levels, hepatic profile, urea and electrolytes were normal and viral antibody testing for Human Immunodeficiency Virus, Hepatitis B Virus and Toxoplasma were negative. Hormone tests including thyroid, growth and bone profile were normal. Genital ambiguity indicated fetal hyperandrogenism, but at age of 4 months serum testosterone was only mildly elevated (0.59 nmol/L, RR: 0.1-0.38 nmol/L). Dehydroepiandrosterone-sulphate and 17-hydroxyprogesterone were also slightly elevated (2.75 µmol/L, Reference Range: 0.14-1.68 µmol/L; 5.96 nmol/L, Reference Range: 0.57-4.81 nmol/L, respectively), but androstenedione levels were normal (3.49 nmol/L, RR: 0.03-6 nmol/L;). 11-deoxycortisol was very high (46.6 nmol/L, RR: < 7.56 nmol/L), suggesting a genetic defect in steroid 11-hydroxylase (*CYP11B1*). Basal plasma cortisol levels were 270.3 nmol/L with a mildly increased ACTH (31.9 pmol/L), confirmed in another sample with higher levels of ACTH (90.9 pmol/L), indicating possible compensated adrenal insufficiency. Basal luteinizing hormone (LH) and follicle-stimulating hormone (FSH) were in the normal range for her age (3.7 mIU/ml and 7.8 mIU/ml,

respectively). Molecular genetics of the most frequent steroidogenic disorders associated with ambiguous genitalia were performed, being negative for *CYP21A2* (MIM: 613815), *CYP11B1* (MIM: 610613) and *POR* (MIM: 124015). At the age of 5 months, she was hospitalized in Pediatric Intensive Care Unit due to acute respiratory failure after a viral infection. She developed a few episodes of central apnea, requiring invasive mechanical ventilation and presented severe hypoglycemia (1.2 mmol/l) with normal electrolytes. She was on treatment with stress dose steroids because of suspected adrenal insufficiency and antihypertensive treatment due to increased blood pressure. The cardiovascular examination was normal. Brain MRI showed delayed myelination, cerebral atrophy and thinning of the corpus callosum. An extensive metabolic screening was negative for an underlying cause. Muscle biopsy showed mild variation in the size of the muscle fibers without structural alterations or pathological deposits and no specific mild increase in cytochrome c oxidase activity without "red ragged" or cytochrome c oxidase-negative fibers. Respiratory chain in muscle and fibroblasts biopsy and mitochondrial DNA investigations were normal. Other neuromuscular studies including electromyography were normal. Progressively she presented a severe and irreversible encephalopathy with chronic respiratory failure, swallowing difficulties, and other abnormal neurological functions and finally she passed away at 9 months.

Sibling B. The second girl of this family was born at 37-0/7 weeks by normal delivery in Equatorial Guinea, two years after her sister. Apgar scores was 9/10 and her birth weight was 2.840 g. Pregnancy was unremarkable and the mother was on carbamazepine treatment. Sibling B presented with global developmental delay with choreiform movements of head and upper limbs, bilateral hearing loss and blindness. She had undergone a genital surgery to repair her ambiguous genitalia in another country. She was admitted at 4 months in a hospital in Spain because of an acute respiratory infection. Her weight was 7 Kg (-0.1 SD), height 59 cm (-1.5 SD) and head circumference 40 cm (-1.5 SD). Physical examination revealed systolic blood pressure in the normal to high range for age. Karyotyping, FISH *SRY* and microarrays were normal (46, XX). Initially, congenital adrenal hyperplasia (CAH) was suspected, but electrolytes and urinary sodium were normal. At birth 17-hydroxyprogesterone was 13.31 nmol/L. Blood tests including full blood count, hepatic profile, urea and electrolytes, growth, bone and thyroid profile and viral antibody testing were normal. Like her sister, ophthalmology review found bilateral cataracts and optic nerves atrophy and abdominal and pelvic ultrasounds revealed hydrocolpos with female internal genitalia. During her admission, she had few episodes of central apnea. MRI of brain showed hypomyelination and brain atrophy and electroencephalogram was normal. Metabolic screen including amino acids, organic acids, acylcarnitine and sterol profile, carbohydrate-deficient transferrin and transferrin isoform analysis, galactitol, galactonate and

galactose-1-phosphate were normal. Ammonia, lactate, creatine kinase and blood gas were normal. Mitochondrial enzyme studies in fibroblasts were also normal. Levels of testosterone, dehydroepiandrosterone sulfate, and 17-hydroxyprogesterone were slightly high (1.35 nmol/L, RR: 0.1-0.38 nmol/L; 5.5 µmol/L, RR: 0.13-1.68 µmol/L; 11.8 nmol/L, RR: 0.57-4.81 nmol/L; respectively) with normal androstenedione (4.43 nmol/L, RR: 0.03-6 nmol/L). Basal plasma cortisol was in the lower range (187.5 nmol/L) with high adrenocorticotropin levels (59.9 pmol/L). Luteinizing hormone and follicle stimulating hormone and estradiol were normal. Human chorionic gonadotrophin stimulation test showed an absent response 72 hours after administration. At 5 months she developed a severe respiratory depression with hypercapnia requiring invasive mechanical ventilation. Chest X-ray, echocardiogram and electrocardiogram were normal. Vasoactive or inotropic drugs were not required. Progressively, she presented neurological impairment with fluctuating levels of consciousness, choreiform movements and absent trunk and tendon reflexes without sedation drugs. Acute phase proteins were normal, with negative test for respiratory viruses and sterile blood culture. Urine culture yielded *Escherichia Coli* and *Citrobacter Koseri* ESBLs, which were treated with antibiotics. Stool culture yielded *Salmonella spp*., interpreted as a past infection because of absent of signs of disease. Finally, after 14 days in pediatric intensive care unit she passed away. An autopsy was performed. Macroscopically, the only remarkable finding was a mild increase in adrenal glands conjoint weight (7.1 g; N 4.8 ±2.2 gr); histology presented mild signs of cytoplasmic vacuolization, possibly due to lipid deposition (Figure S3). Specifically, none of the findings usually associated with CAH, like depletion of the lipid-rich cells of the zona fasciculata, was found. Internal genitalia consisted of a uterus, Fallopian tubes, and ovaries; microscopic examination showed no anomalies. The ovarian cortex displayed a normal population of primordial follicles, with some of them maturing towards secondary and tertiary follicles. As for central nervous system, there was extensive astrogliosis and discrete vacuolization of the white matter, with mild lymphocytic inflammation in perivascular spaces and leptomeninges, and microglial activation.

Expression profiling of FDXR, FDX1 and FDX2 in reprogrammed, induced adrenal-like cells (iALC)

RNA was extracted from wild-type and variant iALC using TRI Reagent (Sigma) and the Direct-zol miniprep RNA kit (Zymo Research), following the manufacturer's protocol. RNA was reverse transcribed using the High-Capacity cDNA Reverse Transcription Kit (Thermo Fisher Scientific). Gene expression analysis was performed by real-time quantitative PCR using the PowerUp SYBR Master Mix and the QuantStudio 1 thermocycler (Thermo Fisher Scientific) and target gene specific primers. Technical triplicates were used to minimize variability. Transcripts encoding beta actin were used as internal control, and data were expressed using the 2 (−ΔΔCt) method.

In silico analysis

We built a homology model of human FDXR and analyzed the sequence and structural changes caused by mutations in *FDXR* indicated in Figure 3. Multiple cross-species sequence alignment showed that the mutations identified in our index patients and iPSC cells are in highly conserved residues, indicating that these amino acids may be significant to the structure or function of the protein (Figure 3B). In addition, the evolutionary conservation analysis indicated that all patient mutations are found in highly conserved amino acids (data now shown). The sequence conservation results were confirmed quantitatively by PolyPhen-2 analysis, which indicated that the G437R amino acid change was probably damaging, with a score of 0.975 (sensitivity: 0.76; specificity: 0.96), and the R386W mutation was also predicted to be damaging, with a score of 1.0 (sensitivity: 0.00; specificity: 1.00) (Table S2). The other mutations were also predicted to be 'probably damaging' under PolyPhen-2 analysis (F51L: score 0.968, sensitivity: 0.77; specificity: 0.95; P74L: score 1.0, sensitivity: 0.00; specificity: 1.00; R155W: score 1.0, sensitivity: 0.00; specificity: 1.00; R193H score 1.0, sensitivity: 0.00; specificity: 1.00) (Table S2).

We then analyzed the impact on structural stability of mutated FDXR proteins compared to WT FDXR (Figure 3C and D). The G437 residue is located within the predicted ferredoxin reductase-type FAD-binding domain, based on computer-generated annotations derived from UniProt sequences of human FDXR. The replacement of G437 by arginine is predicted to disrupt multiple interactions with R435 and I441 residues (Table S2), perturbing the structural stability of the enzyme. The same destabilizing effect was predicted by DynaMut (-0.99 kCal/mol), mCSM (-0.93kCal/mol) and DUET (-1.23 kCal/mol). A calculation of vibrational entropy energy difference between WT and G437R mutant proteins showed a $\Delta\Delta$Svib of -0. 6 kCal^{-1}.mol.K^{-1} indicating a reduction in molecular flexibility of the mutant protein. Disruption to the structural stability of the enzyme is expected to lead to a change in interaction with FDX and other partner proteins (1) and would therefore impact the activities of mitochondrial cytochromes P450 that require FDX as their redox partner (2). Based on the computer-predicted annotation of the human FDXR in UniProt, the R386 residue is in a flexible loop within the NADP binding region (Figure 3A). Stability calculations predicted that the replacement of R386 by W also disrupts multiple atomic interactions within FDXR (D424, M420, Y394, M388, N382) (Table S2). Consequently, an increased rigidity in structure of the R386W mutant FDXR was predicted (Figure 3D). All prediction methods indicated a slight increase in protein stability (DUET -0.17 kCal/mol, mCSM -0.23 kCal/mol, and DynaMut 0.1 kCal/mol) (Table S2). Calculation of vibrational entropy energy difference between WT and R386W mutant proteins showed a $\Delta\Delta$Svib of –0.06 kCal^{-1}.mol.K^{-1} indicating a slight decrease in molecular flexibility (Table S2). Among the other mutations discussed in

this report, F51L and R193H were predicted to cause increased flexibility in parts of the protein and P74L and R155W were predicted to have increased rigidity in overall structure (Figure 3D).

Supplementary Figures and Figure Legends

Figure S1

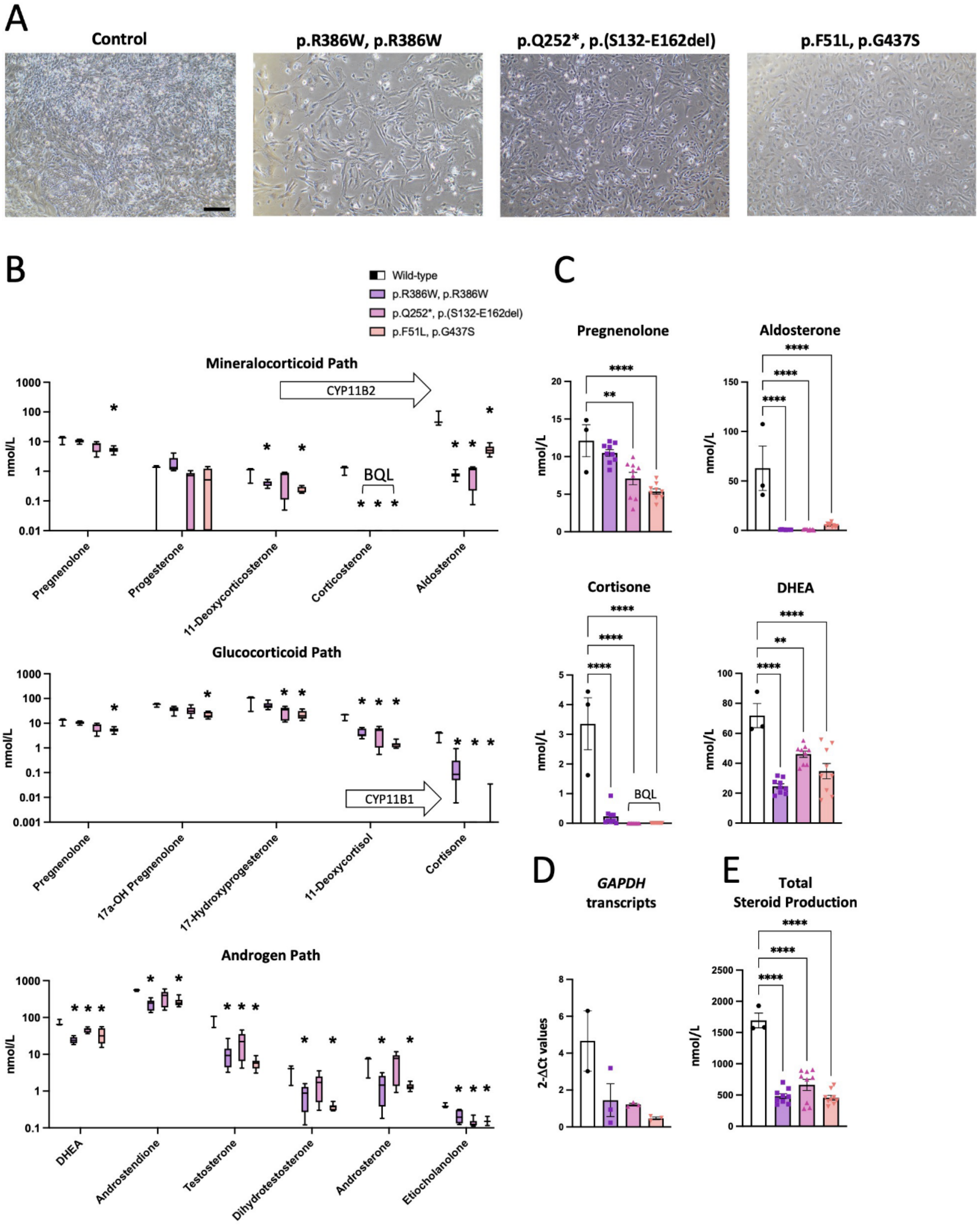

Figure S1. Adrenal-like cells from FDXR patients display lower production of all three steroid categories and are reprogrammed less efficiently than control cells. (A) Representative micrographs of reprogrammed patients' and control individual's fibroblasts at day 20 following differentiation start in induced pluripotent cells. Acquisition was carried out using a 20x objective; scale bar = 150 um. **(B)** Steroid amounts in culture media secreted by reprogrammed fibroblasts from FDXR patients compared to control values (representing steroid amounts of reprogrammed fibroblasts from a non-affected individual). Steroids are split among three graphs according to their belonging to a specific steroid class. BQL, Below Quantification Level, indicates the samples in which steroid levels were not measurable above the lowest quantification limit using LC-MS. Asterisks reflect discoveries found using a multiple unpaired t-test assuming individual variance for each steroid. **(C)** Concentrations as raw values of the endpoint or critical steroids for each pathway using a linear scale. **(D)** Transcript levels for the normalizer *GAPDH* calculated with the $2^{-\Delta Ct}$ method for each reprogrammed cell line. **(E)** shows the total levels of steroids detected using LC-MS within each cell line. Statistical analysis was conducted using a one-way ANOVA analysis followed by a Dunnett's multiple comparisons test.

Figure S2

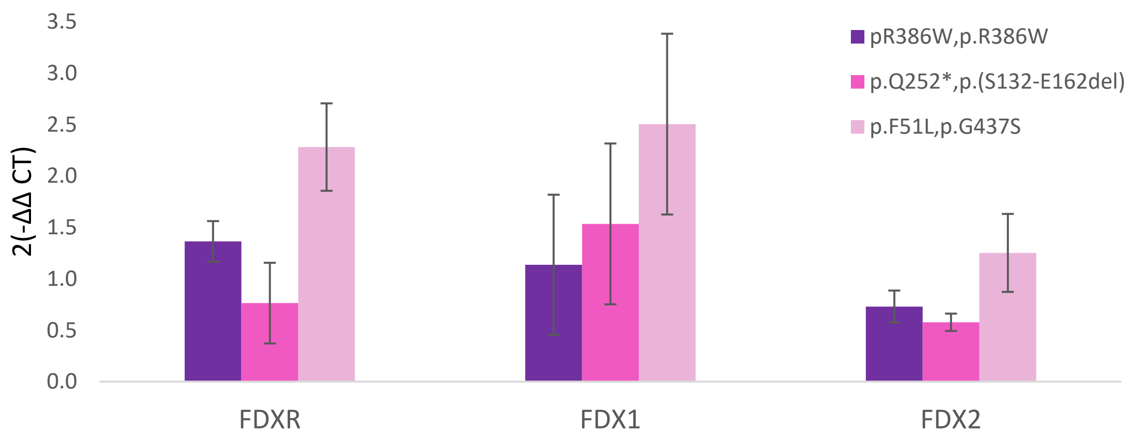

Figure S2. FDXR, FDX1 and FDX2 expression in reprogrammed adrenal-like cells from fibroblasts of three patients with FRM. Transcript levels of specific genes in variant iALC are displayed in relation to wild-type control iALC, normalized to beta actin expression. Data are expressed using the 2 (−ΔΔCt) method. Mean and SDS of three experiments is shown.

Figure S3

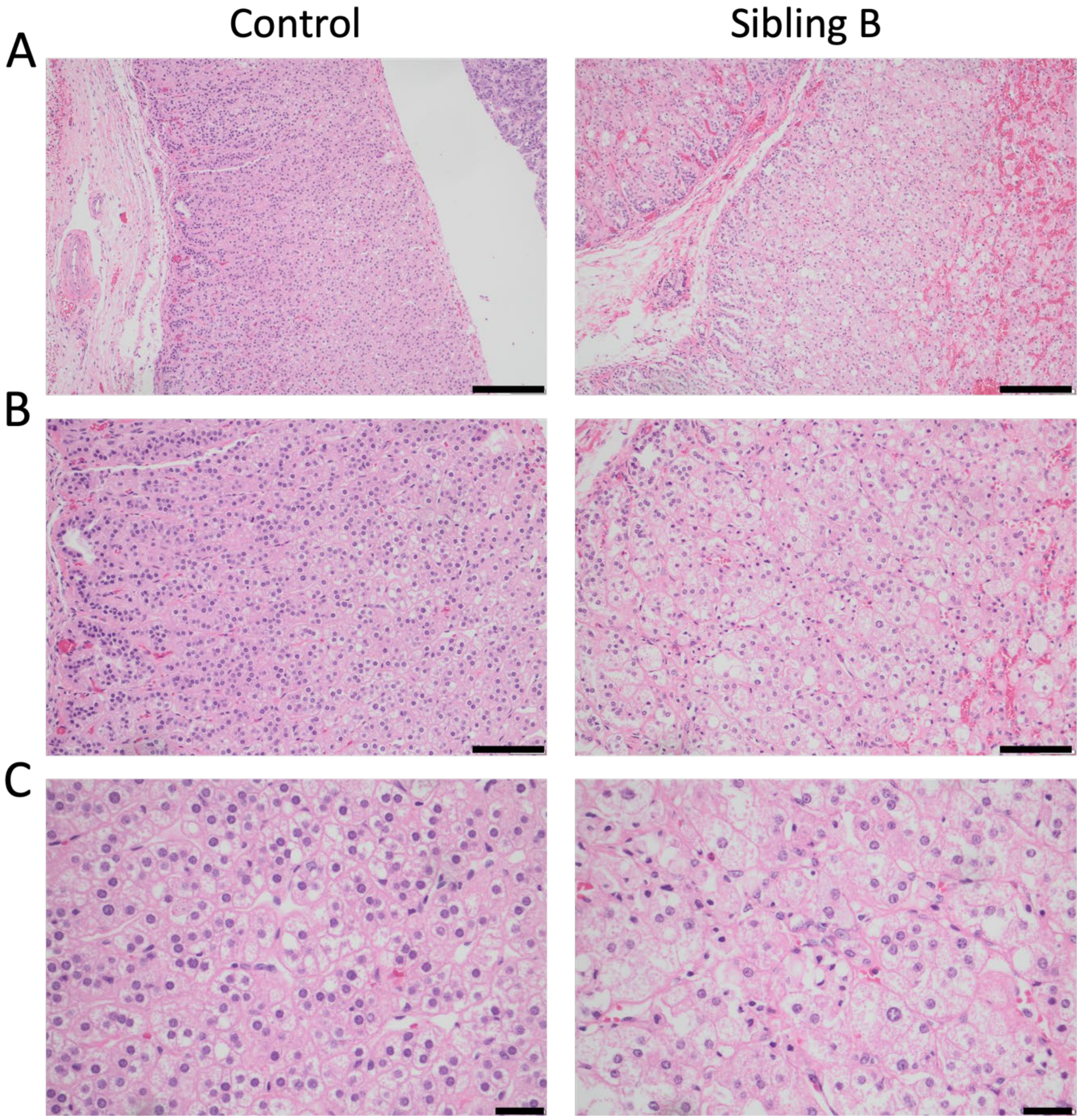

Figure S3. Histological micrographs of the adrenal cortex of index patient B (Sibling B) in comparison to a healthy control. Representative pictures of haematoxylin-eosin-stained sections were captured at 10x **(A)**, 20x **(B)**, and 40x **(C)**. Scale bars indicate 200μm **(A)**, 100μm **(B)**, and 40μm **(C)**. Note the diffused cytoplasmic vacuolization in Sibling B's adrenal cortex.

Supplementary Tables

Table S1. Review of published FDXR-related neuropathy patients as of June 2023, with the addition of two index cases described in this work.

**Biological Sex**	**Protein Variant**	**DNA Variant**	**Age at last examination (yr)**	**Known FRM phenotypic features** (age at diagnosis)<br>O: Ophthalmological features<br>H: Hearing<br>CNS: Central nervous system features<br>PNS: Peripheral neuropathy<br>Add: additional	**Clinical hints for adrenal insufficiency** (age at event(s)/diagnosis)	**Sudden death (yr)**	**Ref**
M	p.R193C, p.P372H	c.577C>T, c.1115C>A		O: OA, N, S (3yr)<br>CNS: A, dizziness, tremor (12yr)			4
M[f]	p.R309*, p.P372H	c.925C>T, c.1115C>A		O: Nyctalopia (4yr), macular edema (5yr), cataracts, bilateral optic atrophy, retinal vessel attenuation, peripheral pigmentary changes (11yr)<br>H: impaired (5yr)<br>CNS: Verbal dyspraxia (childhood)<br>PNS			4
M[f]	p.R309*, p.P372H	c.925C>T, c.1115C>A		O: OA, retinal dystrophy, macula edema, retinal vessels attenuation, constricted visual field, nyctalopia (14yr)<br>H: Hearing loss (14yr)<br>CNS: A<br>PNS			4
M	p.A154V, p.A275W	c.461C>T, c.823C>T		O: OA, retinal dystrophy (7yr), retinal dysfunction (8yr), and progressive loss of retinal vessels, N (14yr), cataract (18yr)<br>H: loss (4yr)<br>CNS: Tremor (15yr), learning difficulties			4
M	p.P372H, p.A427P	c.1115C>A, c.1279G>C	6	O: atrophic pale optic nerve (24yr)<br>PNS	Shortness of breath and low exercise tolerance		4
F	p.T205M, p.R275W	c.614C>T, c.823C>T		O: OA (40yr)<br>H: Mild deafness (childhood), hearing loss (40yr)<br>CNS: Learning difficulties, clumsiness (childhood)<br>PNS			4

M	p.P372H	c.1115C>A, c.chr17:74818633 – 74888183del		O: Retinitis pigmentosa (7yr), N, OA (later in childhood), cataracts (17yr)<br>PNS: A (infancy), progressive in association with viral infections and fever. Attention deficit hyperactivity disorder (17yr)<br>PNS	Progression of disease of after febrile illnesses (childhood)		4
M[f]	p.P372H, p.G397S	c.1115C>A, c.1189G>A		O: OA and rod cone dystrophy (2.5yr)<br>CNS: Absent ankle jerks, impaired joint position, and vibration sense and gait A (12yr)<br>PNS			4
F[f]	p.P372H, p.G397S	c.1115C>A, c.1189G>A		O: OA and retinal dystrophy (3.5yr)<br>CNS: Gait A, brisk knee jerks, absent ankle jerks, and impaired vibration sense to the knee (15yr)<br>Cyclical vomiting (15yr)			4
F	p.R242W, p.R306C	c.724C>T, c.916C>T		O: OA (42yr)<br>H: loss (42yr)<br>PNS	Vomiting, constipation, and severe weight loss (42yr)		4
F[f,c]	p.R306C, p.R306C	c.916C>T, c.916C>T	6	O: OA (13yr)<br>H: BAN (13yr)			5
F[f,c]	p.R306C, p.R306C	c.916C>T, c.916C>T	38	O: OA (13yr)<br>H: BAN (13yr)			5
F[f]	p.R306C, p.R306C	c.916C>T, c.916C>T	36	O: Infraclinical OA (36yr)<br>H: BAN (15yr)			5
F[f]	p.R306C, p.R306C	c.916C>T, c.916C>T	31	O: Infraclinical OA (11yr)<br>H: BAN (11yr)			5
M	p.R306C, p.Q419*	c.916C>T, c.1255C>T	31	O: Infraclinical OA (31yr)<br>H: BAN (teenage)			5
M	p.R242W, p.R327S	c.724C>T, c.979C>A	20	O: Bilateral retinitis pigmentosa (2yr), OA (20yr), and<br>H: BAN (20yr)<br>CNS/PNS: Hypopallesthesia of the lower limbs (20yr)			5
F[f]	p.L215V, p.E477K	c.643C>G, c.1429G>A		O: OA (childhood)<br>H: BAN (17yr)<br>CNS: Slight language delay			5
F[f]	p.L215V, p.E477K	c.643C>G, c.1429G>A		O: OA (17yr)<br>H: BAN (5yr)			5

M	p.F51L, p.G437S	c.151T>C, c.1309G>A	14	O: Bilateral retinitis pigmentosa (7yr), likely OA (blindness - 14yr)<br>CNS: Spasticity, A<br>PNS	Gait deterioration following acute illness (after 8yr)		3
M[f]	p.R386W, p.R386W	c.1156C>T, c.1156C>T	1.6	O: Poor visual tracking and S (0.3yr),<br>CNS: Delayed motor development, hypotonia (0.3yr), abnormal corpus callosum (0.7yr). GDD; restricted diffusion of the globi pallidi and the midbrain, central and cortical atrophy (0.9yr). Non progressive cortical atrophy (1.4yr)<br>Mitochondrial dysfunction and dysmorphism (postmortem)	Hypotonia.<br>Chronic respiratory failure (1yr).<br>Death after febrile illness.	1.6	3
F[f]	p.R386W, p.R386W	c.1156C>T, c.1156C>T	1.4	O: OA (0.4yr)<br>CNS: Delayed myelinization (0.4yr), GDD (1.3yr), regression, hypotonia.<br>Born at 39wk of gestation following maternal preeclampsia.			3
M	p.R386W, p.R386W	c.1156C>T, c.1156C>T	6	O: OA (1.6yr)<br>CNS: Microencephaly, GDD, regression, hypotonia, spasticity, movement disorder.<br>Born at 32wk of gestation following maternal preeclampsia.<br>Gastroesophageal reflux, sleep disturbances, mild joint contractures, and one café-au-lait spot (childhood). Elevated citrate synthase levels.	Respiratory failure and demise following influenza infection (6yr)	6	3
M	p.R386W, p.R386W	c.1156C>T, c.1156C>T	2.3	O: OA and S (1.5yr)<br>H: Abnormal auditory brainstem responses (1.5yr)<br>CNS: Decreased corpus callosum, abnormal frontal lobe, diffuse cerebral dysfunction.<br>Failure to thrive (0.6yr)	Hypoglycemia episode that required ICU admission (2yr)	2.3	3
F	p.R386W, p.P74L	c.1156C>T, c.221C>T	4	O: OA (4yr)<br>CNS: Microencephaly, increased cerebral atrophy, decreased cerebral white matter, basal ganglia, globi pallidi and substantia nigra, severe encephalopathy, GDD, regression, hypotonia, spasticity (4yr)	Poor weight gain, recurrent ear and urinary tract infections, difficulty swallowing, muscle weakness, constipation, scoliosis, loose-joints, irritability and<br>excessive sleepiness, abnormal mitochondrial functionality (4yr). Increased citrate synthase activity.<br>Hypoventilation, moderately increased body hair		3

M	p.V158M, p.K274*	c.472G>A, c.820A>T	32	O: N (0.5yr), OA (2.5yr)<br>H: loss (12yr)<br>CNS: Abnormal gait, lack of speech development (2yr). Seizures, abnormal EEG, GDD, hypotonia, spasticity, A (2.5yr). Cerebral and cerebellar atrophy, gliosis (postmortem).<br>PNS<br>Liver biopsy demonstrated hepatocytes with PAS positive inclusions (2.5yr)	Persistent respiratory illness (wheezing, coughing - childhood)	32	3
F	p.R309*, p.P403L	c.925C>T, c.1208C>T	4	O: S, likely OA (1yr)<br>CNS: Swelling of the cerebellum, GDD, hypotonia, A (2yr, following fever and rash)	Loss of developmental skills and presentation of ataxia following fever and rash (2yr)		3
M	p.V158M, p.I213F	c.472G>A, c.637A>T	16	O: OA (3.5yr), retinitis pigmentosa<br>H: Moderate sensorineural hearing loss (0.8yr)<br>CNS: GDD, regression, hypotonia, spasticity, Babinski signs, poor balance, unsteady gait with deterioration. Loss of balance and bladder control after a respiratory infection (7yr). Atypical anxiety disorder, developmental<br>expressive language disorder, cognitive progressing disorder, and fine motor apraxia (18yr).<br>PNS<br>Febrile seizure (1.2yr). Mitochondrial accumulation, elevated citrate synthase activity (4yr).<br>Temporally lost balance and bladder control after a respiratory infection (7yr).			3
F[f,c]	p.D368N, p.D368N	c.1102G>A, c.1102G>A	10	O: OA (8yr, following viral infection events), N<br>H: loss (8yr, following viral infection events)<br>CNS: Motor and sensory neuropathy, microencephaly, speech difficulties, impaired cognition, bilateral pain in lower limbs, muscle weakness.	Recurrent infections. Cognitive loss following encephalopathy. Temporary loss of ambulatory skills following a 'flu-like' episode. Muscle weakness.		3
F[f,c]	p.D368N, p.D368N	c.1102G>A, c.1102G>A	7	O: OA (4yr, following viral infection events), N<br>H: loss (4yr, following viral infection events)<br>CNS: Motor and sensory neuropathy, microencephaly, speech difficulties, impaired cognition, bilateral pain in lower limbs, muscle weakness.	Recurrent infections. Cognitive loss following encephalopathy. Temporary loss of ambulatory skills following a 'flu-like' episode.		3

M	p.T211A, p.G437C	c.631A>G, c.1309G>T	3	O: OA (~0.5yr)<br>CNS: Microencephaly, cerebral atrophy, abnormal white matter paraventricular signal, seizures, EEG with chaotic background with spasm, GDD, regression, severe hypotonia.<br>Marginal loss of mitochondrial respiratory chain complex IV activity in muscle biopsies.	Deterioration after upper respiratory tract infection (0.9yr)		3
F[f]	p.C353Y, p.I143F	c.1058G>A, c.427A>T	4	O: OA (1.5yr)<br>CNS: Abnormal basal ganglia and mesencephalic peduncles, GDD, regression, spasticity	Regression of motor milestones and progressive encephalopathy disease with quadriplegia and GDD following a febrile episode (0.3yr). Clinical worsening following another febrile episode (1.5yr).		3
M[f]	p.C353Y, p.I143F	c.1058G>A, c.427A>T	2	CNS: Neonatal hypertonia, microcephaly, epilepsy, and GDD		2	3
M	p.R386W, p.R386W	c.1156C>T, c.1156C>T	6	O: Likely OA (6yr)<br>CNS: GDD, hypotonia, stereotypical hand movements, disconjugate gaze, and nonreactive pupils (1.8yr)<br>Pyloric stenosis			3
M[f,c]	p.R386W, p.R386W	c.1156C>T, c.1156C>T	1.8	O: OA and retinitis pigmentosa (childhood)<br>CNS: GDD, regression, hypotonia, spasticity, encephalopathy<br>Dysphagia	Apnea following infection (0.8yr)		3
M[f,c]	p.R386W, p.R386W	c.1156C>T, c.1156C>T	2.8	Demise due to acute cardiopulmonary arrest		2.8	3
M	p.M1V, p.R155W	c.1A>G, c.463C>T	2.8	O: OA, N (2yr)<br>CNS: A (2yr), ataxic gait, axial hypotonia, GDD (speech delay, fine and gross motor skills)<br>Born at 29 weeks because of maternal preeclampsia.	Deterioration after febrile respiratory infection (2yr)		6

F	p.S310Tfs*19, p.R193H	c.929delG, c.578G>A	0.9	O: OA (0.2yr),<br>CNS: Normal pituitary, thin optic nerves (0.2yr), cerebellar vermis hypoplasia (0.2yr) and bilateral cataracts (0.3yr). Diminished size of the optic nerves and chiasm and global cerebral atrophy (0.6yr). Muscular spasticity, depletion of Purkinje cells, lack of lower motor neurons, gliosis (postmortem).<br>Born at 30 weeks because of maternal hypertension. The patient was hospitalized due to lethargy, Klebsiella urosepsis and enteroviral meningitis (0.6yr). During her hospitalization, she had a few episodes of apnea and experienced an anoxic event. At 0.7yr, the patient developed an acute respiratory failure and was intubated with supportive care.	Episodic apnea, failure to thrive, GDD, and episodic apnea following Klebsiellaurosepsis and enteroviral meningitis (0.6yr).<br>Poor feeding, abdominal distension, and respiratory distress (at birth). Acute respiratory failure (0.7yr).<br>Labial fusion and clitoromegaly, with 46,XX genotype. Gonadal biopsy showed immature ovarian parenchyma bilaterally.	0.9	6
F	p.S12W, p.R228L	c.35C>G, c.683G>T		O: OA<br>H: impairment<br>CNS/PNS: Distonia, A, polyneuropathy, basal ganglia lesions. Psychiatric disorder.			7
M	p.V111A, p.L189_A192 del	c.332T, c. 565-576del	0.9	O: OA, N (0.6yr)<br>CNS: Myopathy, GDD, seizures, basal ganglia lesions, infection related deterioration, raised cerebrospinal fluid lactate, respiratory insufficiency, Leigh syndrome (0.6yr)<br>Diabetes mellitus, anemia, respiratory insufficiency	Sudden death: Epileptic seizures, loss of consciousness, and loss of brain stem reflexes including respiratory drive and blood pressure regulation following high fever episode (0.9yr)	0.9	7
F	p.G109S, p.R448Q	c.325G>A, c.1343G>A	3.5	H: impairment<br>CNS: Microcephaly, GDD, movement disorder, A			7
M	p.G109S, p.R448Q	c.325G>A, c.1343G>A		CNS: Microcephaly, movement disorder, A			7
M[f]	p.R228L, p.C353Y	c.683G>T, c.1058G>A	1.4	O: Bilateral nuclear cataracts (0.2yr), OA<br>H: loss (0.2yr)<br>CNS: Microcephaly, movement disorder, A, infantile onset progressive encephalopathy with delayed myelination (0.5yr)	Infection-related deterioration<br>Apnea and loss of consciousness (0.5yr)<br>Cryptorchidism and micropenis	1.4	7
M[f]	p.R228L, p.C353Y	c.683G>T, c.1058G>A	0.5	O: Bilateral nuclear cataracts (0.2yr), OA<br>H: loss (0.2yr)<br>CNS: Microcephaly, movement disorder, A, infection-related deterioration, infantile-onset progressive encephalopathy with delayed myelination	Cardiorespiratory failure leading to death (0.5yr)	0.5	7

F	p.A211V, p.A211V	c.632C>T, c.632C>T	4	O: OA<br>H: impairment<br>CNS: regression, spasticity, A, cerebral atrophy, and cerebellar atrophy			7
F	p.R386W, p.R386W	c.1156C>T, c.1156C>T		O: OA, S<br>CNS: Hypotonia, GDD, nonambulatory			7
M	p.P84S, p.D206H	c.250C >T, c.616G>C	1	O: OA (0.7yr)<br>H: loss (0.2yr)<br>CNS: GDD, hypotonia, motor retardation (0.6yr)<br>Born at 36 weeks of gestation. Elevated homocysteine, lactate, AST, ALT, ammonia (0.7yr).	Sudden death after fever episode associated with sepsis and septic shock (1yr)	1	8
F	p.D368N, p.V314L	c.1102G>A, c.940G>T	25	O: OA (12yr)			9
M[f]	p.R155W, p.R155W	c.463C>T, c.463C>T	14	O: OA (13yr)<br>H: BAN (11yr)<br>CNS: A, hypotonia (11yr)<br>PNS: Lower limb pain (11yr)<br>Nocturnal enuresis (9yr)	Partial recovery from A and peripheral neuropathy after glucocorticoid treatments. Amelioration of sensitive symptoms and partial recovery of autonomous gait following IV methylprednisolone (11yr)		10
F[f]	p.R155W, p.R155W	c.463C>T, c.463C>T	9	O: OA (7yr)<br>CNS: Flaccid paralysis with loss of trunk control, and autonomous ambulation (7yr).<br>PNS: Mild dysarthria, diffuse hypotonia, upper limb hyperreflexia and proximal weakness, lower limb areflexia and flaccid paralysis, dysesthesia, and pain in distal regions. T2 hyperintensities in the posterior columns and posterior root contrast enhancement (7yr).	Severe abdominal pain (7yr)		10
F[f,c]	p.R193H, p.R193H	c.578G>A, c.578G>A	1.1	O: OA (0.8yr)<br>Born prematurely at 34 weeks (twin)	Died following severe pneumonia (1.1yr)	1.1	11
F[f,c]	p.R193H, p.R193H	c.578G>A, c.578G>A	1.5	O: OA (<1yr)<br>Born prematurely at 34 weeks (twin)	Died following severe respiratory infections due to hand, foot, and mouth disease (1.5yr)	1.5	11
F	p.R79C, p.R104C	c.235C>T, c.310 >T	8	O: OA (2yr)<br>CNS: Mild mental retardation (8yr)			11
F	p.G134R, p.R306H	c.400G>A, c.917G>A	3	O: OA (1yr)<br>CNS: Microcephaly, hypertonia, and developmental delay (0.8yr). A, and cerebellar atrophy (2yr).	Febrile convulsions (0.8yr)		11

F	p.R115G,<br>p.Q233R	c.463C>T,<br>c.698A>G	16	O: OA (6yr)<br>Muscle weakness in the lower extremity (infancy).			11
M	p.R228W,<br>p.V314L	c.682C>T,<br>c.940G>T	16	O: OA (16yr)			11
M	p.R104C,<br>p.R104C	c.310C>T,<br>c.310C>T	19	OA (19yr)			11
M	p.R115G,<br>p.G325D	c.343A>G,<br>c.974G>A	16	OA (12yr)			11
M	p.L232P,<br>p.R275W	c.695T>C,<br>c.823C>T		O: OA (6yr)	Deterioration after fever (5yr)		11
F	p.R104C,<br>p.W398C	c.310C>T,<br>c.1194G>T		O: OA (4yr)			11
M	p.R79C,<br>p.V314L	c.235C>T,<br>c.940G>T		O: OA (7yr)			11
F	p.R79C,<br>p.V314L	c.235C>T,<br>c.940G>T		O: OA (5yr)			11
F[f]	p.R155W,<br>p.R155W	c.463C>T,<br>c.463C>T		O: OA (6yr)<br>CNS: A, GDD<br>PNS<br>Muscle atrophy, weak tendon and muscle reflexes, dystonia			12
M[f]	p.R155W,<br>p.R155W	c.463C>T,<br>c.463C>T		O: OA (?)			12
F	p.R386W,<br>p.R386W	c.1156C>T,<br>c.1156C>T		O: OA, vision loss, S<br>CNS: GDD, regression, hypotonia<br>PNS: Movement disorder, spasticity<br>Iron staining on teeth			13
M	p.P403L,<br>p.R386W	c.1208C>T,<br>c.1156C>T		O: Vision loss, N<br>H: loss<br>CNS: GDD, regression, hypotonia, cerebellar degeneration<br>PNS: Movement disorder			13
F	p.R309*,<br>p.P403L	c.925C>T,<br>c.1208C>T		O: OA, vision loss, S, N, retinopathy<br>CNS: GDD, regression, hypotonia, swelling of the cerebellum.<br>PNS: Movement disorder, spasticity			13

F	p.R309*,<br>p.P403L	c.925C>T,<br>c.1208C>T		O: OA, vision loss, N<br>CNS: GDD, regression, hypotonia<br>PNS: Movement disorder			13
F	?, p.G397S	c.1003-6C>G,<br>c.1189G>A		O: OA, vision loss, S, N<br>CNS: GDD, regression, hypotonia, seizures, progressive cerebral atrophy<br>PNS: Movement disorder<br>Hypermobility, headaches			13
F	p.R306C,<br>p.R306C	c.916C>T,<br>c.916C>T		O: OA, vision loss, S<br>H: loss			13
M	p.M1V,<br>p.D368N	c.1A>G,<br>c.1102G>A		O: OA, vision loss, S, N<br>H: loss<br>CNS: Regression, cerebellar degeneration<br>PNS: Movement disorder	Regression treated with prednisone in childhood, psychiatric disturbances		13
M	p.R386W,<br>p.R386W	c.1156C>T,<br>c.1156C>T		O: OA, vision loss, N<br>CNS: GDD, regression, hypotonia, delayed myelination, nonspecific prominence of the ventricles and subarachnoid spaces<br>PNS: Movement disorder			13
F	?, p.R386W	c.279+5G>T,<br>c.1156C>T		O: OA, vision loss, S<br>CNS: GDD, regression, hypotonia<br>PNS: Movement disorder	Hypertension		13
F	p.R386W,<br>p.R386W	c.1156C>T,<br>c.1156C>T		O: OA, vision loss, N<br>H: loss<br>CNS: GDD, regression, hypotonia, seizures, absence of the septum pellucidum, reduced optic apparatus, severe hypoplasia of olfactory apparatus.<br>PNS: Movement disorder, spasticity<br>Hyperammonemia	Hypertension		13
F	p.R155W, ?	c.463C>T,<br>c.802+3G>C		O: OA, vision loss, S, retinopathy<br>CNS: GDD, regression, hypotonia<br>PNS: Movement disorder			13
F	p.R327H,<br>p.L461fs	c.980G>A,<br>c.1382_1392de<br>l insATCC		O: OA, vision loss, retinopathy, cataracts<br>CNS: Thinned optic chiasm<br>PNS: Movement disorder<br>Marfanoid habitus, hypermobility, spinal cord atrophy			13

F	p.R386W, p.G397S	c.1156C>T, c.1189G>A		O: OA, vision loss<br>H: loss<br>CNS: GDD, regression, hypotonia, seizures<br>PNS: Movement disorder, spasticity			13
M	p.Q252*, p.(S132-E162del)	c.754C>T, c.339G>A		O: OA, vision loss, N<br>CNS: GDD, hypotonia, microencephaly<br>PNS: Movement disorder<br>Complex I, IV deficiency			13
F	p.G437R, p.G437R	c.1309G>C , c.1309G>C		O: Bilateral congenital cataracts . Poor visual tracking, N, small optic nerves (0.3yr)<br>H: Bilateral sensorineural hearing loss (0.3yr)<br>CNS: Generalized hypotonia (0.3yr). Severe and irreversible encephalopathy with chronic respiratory failure, swallowing difficulties and other abnormal neurological functions (0.3 - 0.7yr). Born prematurely at 36 weeks due to preeclampsia.	Testosterone, DHEA-s, and 17-hydroxyprogesterone, and ACTH slightly elevated, but normal cortisol. Slightly high blood pressure (0.3yr).<br>Fusion of the labia majora, clitoromegaly and a common urogenital sinus (stage IV of Prader scale). Hydrocolpos (0.3yr).	0.7	This study
F	p.G437R, p.G437R	c.1309G>C , c.1309G>C		O: Blindness (at birth). Bilateral cataracts and optic nerves atrophy (0.3yr).<br>H: Bilateral hearing loss (at birth).<br>CNS: GDD, choreiform movements of head and upper limbs (at birth). Episodes of central apnea. Hypomyelination and brain atrophy (0.3yr). Severe respiratory depression with hypercapnia, neurological impairment with fluctuating levels of consciousness, absent trunk and tendon reflexes (0.4yr).	Testosterone, DHEA-s, and 17-hydroxyprogesterone, and ACTH slightly elevated, but normal cortisol. a Mild increase in adrenal glands conjoint weight, but remarkable histology.<br>Ambiguous genitalia (0.3yr).	0.5	This study

Footnotes: Variants are annotated to the NM_024417.5 and NP_077728.3 references. f= familial cases. c= consanguinity. Variants in red indicate the patients that donated fibroblasts used for reprogramming in this study. A, Ataxia; OA, Optic Atrophy; N, Nystagmus; S, Squint; BAN, Bilateral Auditory Neuropathy; GDD, Global Developmental Disorder. Ca: Campbell et al., 2023 (manuscript submitted for publication). Works from (3-13) are included.

Table S2. Structural analysis and prediction of functional impact of discrete FDXR mutations.

FDXR mutation	Dynamut kcal/mol	ΔΔG DUET kcal/mol	Δ Vibrational Entropy $\Delta\Delta S_{Vib}$ ENCoM kcal.mol^{-1}.K^{-1}	ΔΔG mCSM kcal/mol	Interactions	PolyPhen2 Score
F51L	-0.04	-0.66	0.55 Increased flexibility	-0.60	S46, P48, T53, I67, P74, F75, F96,	Probably damaging, 0.968
P74L	1.19	0.929	-0.467 Decreased flexibility	0.49	P482, M485	Probably damaging, 1
R155W	-0.6	-0.44	-0.62 Decreased flexibility	-0.46	E136, S153, F157, V158, Y362	Probably damaging, 1
R193H	-1.39	-1.17	0.43 Increased flexibility	-1.27	D85, H86, L189, D190, V191, T197, M244	Probably damaging, 1
R386W	-0.1	-0.17	-0.06 Decreased flexibility	-0.23	N382, M388, Y394, M420, D424	Probably damaging, 1 Increased rigidity and in some places
G437R	-0.99	-1.23	-0.6 Decreased Flexibility	-0.93	A125, A439, A440, I441, R435,	Probably damaging, 0.975

Supplementary References

1. Ewen KM, Ringle M, Bernhardt R. Adrenodoxin--a versatile ferredoxin. IUBMB Life. 2012;64(6):506–512.

2. Grinberg AV, et al. Adrenodoxin: structure, stability, and electron transfer properties. Proteins. 2000;40(4):590–612.

3. Peng Y, et al. Biallelic mutations in the ferredoxin reductase gene cause novel mitochondriopathy with optic atrophy. Human Molecular Genetics. 2017;26(24):4937–4950.

4. Jurkute N, et al. Expanding the FDXR-Associated Disease Phenotype: Retinal Dystrophy Is a Recurrent Ocular Feature. Invest Ophthalmol Vis Sci. 2021;62(6):2.

5. Paul A, et al. FDXR Mutations Cause Sensorial Neuropathies and Expand the Spectrum of Mitochondrial Fe-S-Synthesis Diseases. Am J Hum Genet. 2017;101(4):630–637.

6. Slone J, et al. Biallelic mutations in FDXR cause neurodegeneration associated with inflammation. J Hum Genet. 2018;63(12):1211–1222.

7. Stenton SL, et al. Expanding the clinical and genetic spectrum of FDXR deficiency by functional validation of variants of uncertain significance. Hum Mutat. 2021;42(3):310–319.

8. Yang C, et al. Report of a case with ferredoxin reductase (FDXR) gene variants in a Chinese boy exhibiting hearing loss, visual impairment, and motor retardation. Int J Dev Neurosci. 2021;81(4):364–369.

9. Song S-J, et al. Novel biallelic compound heterozygous mutations in FDXR cause optic atrophy in a young female patient: a case report. Int J Ophthalmol. 2021;14(11):1796–1798.

10. Masnada S, et al. FDXR-associated disease: a challenging differential diagnosis with inflammatory peripheral neuropathy. Neurol Sci. 2023;1–7.

11. Yi S, et al. FDXR-Associated Oculopathy: Congenital Amaurosis and Early-Onset Severe Retinal Dystrophy as Common Presenting Features in a Chinese Population. Genes. 2023;14(4):952.

12. Keller N, et al. Genomic variants causing mitochondrial dysfunction are common in hereditary lower motor neuron disease. Human Mutation. 2021;42(4):460–472.

13. Campbell T, et al. Clinical study of ferredoxin-reductase-related mitochondriopathy: Genotype-phenotype correlation and proposal of ancestry-based carrier screening in the Mexican population. Genetics in Medicine Open. 2024;2:100841.
